# Supplementary material for: A Global Screen for Assembly State Changes of the Mitotic Proteome by SEC-SWATH-MS
Source: Cell Syst. 2020 Feb 26;10(2):133–155.e6. doi: 10.1016/j.cels.2020.01.001 (PMC7042714; doi:10.1016/j.cels.2020.01.001)

Q9H0U3 | MAGT1_HUMAN | MAGT1 IAG2 PSEC0084 UNQ628/PRO1244
Monomer MW [kDa]: 38.037 Monomer expected elution fraction: 49

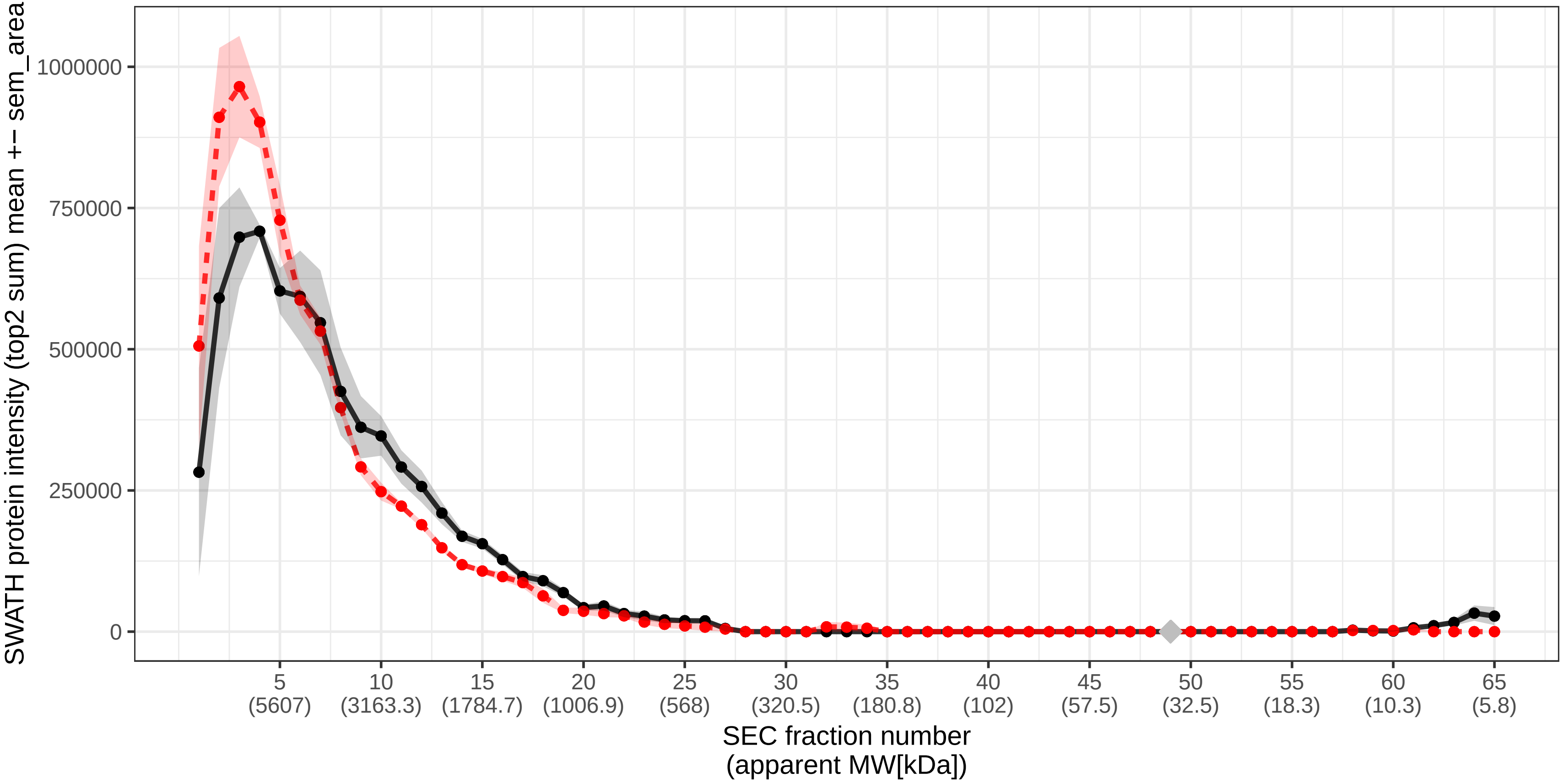

Supplement: Data S1. SEC-SWATH-MS Protein Chromatograms, Related to Figure 1 [file mmc6.zip › SECchrom_Q9H0U3_MAGT1_HUMAN_MAGT1_IAG2_PSEC0084_UNQ628_PRO1.pdf]
